# Phenolic compounds as antioxidants and chemopreventive drugs from *Streptomyces cellulosae* strain TES17 isolated from rhizosphere of *Camellia sinensis*

**DOI:** 10.1186/s12906-018-2154-4

**Published:** 2018-03-09

**Authors:** Riveka Rani, Saroj Arora, Jeevanjot Kaur, Rajesh Kumari Manhas

**Affiliations:** 1 Department of Microbiology, Guru Nanak Dev University, Amritsar, Punjab 143005 India; 2 Department of Botanical and Environmental Sciences, Guru Nanak Dev University, Amritsar, Punjab 143005 India

**Keywords:** Oxidative stress, *Streptomyces cellulosae*, Rhizosphere, *Camellia sinensis*, Antioxidant, Phenolic compounds, Cytotoxicity

## Abstract

**Background:**

Oxidative stress in an intracellular environment created by the accumulation of reactive oxygen species results in oxidative damage to biomolecules which ultimately become a hallmark for severe diseases like cancer, aging, diabetes, and cardiovascular and neurodegenerative diseases.

**Methods:**

Various in vitro assays were employed to assess the antioxidant potential of strain, DNA protective activity was demonstrated using DNA nicking assay and cytotoxicity of the extract was evaluated using MTT assay. Further identification of the compounds was done using UPLC analysis.

**Results:**

The extract of *Streptomyces cellulosae* strain TES17 demonstrated significant antioxidant activity with percentage inhibition of 78.47 ± 0.23, 91.08 ± 0.98 and 82.08 ± 0.93 for DPPH, ABTS and superoxide radical assays at 5 mg/mL, respectively. Total antioxidant and reducing power were found to be 76.93 ± 0.76 and 231.96 ± 0.51 mg AAE/100 mg of dry extract, respectively. Moreover, the extract was shown to inhibit lipid peroxidation upto 67.18 ± 1.9% at 5 mg/mL. TPC and TFC measured in the extract was 55 mg GAE/100 mg and 11.17 ± 4.05 mg rutin/100 mg, respectively. The protective nature of the TES17 extract to oxidative stress induced damaged DNA was shown by percentage of supercoiled DNA i.e. Form I was increased from 26.38 to 38.20% at concentrations ranging from 2 μg to 10 μg. TES17 extract also showed the cytotoxic activity against lung cancer cell line with 74.7 ± 1.33% inhibition whereas, limited toxicity was observed against normal cell line with percentage viability of 87.71 ± 6.66 at same concentration (30 μg/mL) tested. The antioxidant capacity of extract was well correlated with its TPC and TFC and this in turn was in keeping with the UPLC analysis which also revealed the presence of phenolic compounds that were responsible for the antioxidant and cytotoxic potential of *S. cellulosae* strain TES17.

**Conclusions:**

The present study describes that *S. cellulosae* strain TES17 isolated from the rhizosphere of *Camellia sinensis* (tea) plant; produces potent compounds with antioxidant activity, further might be developed into therapeutic drugs to combat oxidative stress.

**Electronic supplementary material:**

The online version of this article (10.1186/s12906-018-2154-4) contains supplementary material, which is available to authorized users.

## Background

Reactive Oxygen Species (ROS) are constantly generated as a byproduct during the electron transport chain in mitochondria [1], the bane to all aerobic species. It is plausible that at low concentration, ROS play an important role in cell signaling including apoptosis and gene expression [2]. A balance between production and removal of ROS is paramount to the survival of all aerobic life forms. However, the disequilibrium of oxidation status due to accumulation of free radicals creates an oxidative stress in intracellular milieu [3]. Oxidative stress has been implicated in physiological aging [4], diabetes [5], development of neurodegenerative diseases such as Parkinson’s disease and Alzheimer’s disease [6], cardiovascular diseases [7] and cancer [8].

In order to antagonize the oxidative stress, antioxidants play an important role in the survival of aerobic species. Antioxidants act as electron donors to the highly reactive species and make them stable [9]. Synthetic antioxidants such as butylated hydroxyanisole (BHA), butylated hydroxytoluene (BHT) and propyl gallate (PG) have been used but their use is being restricted due to their low solubility, moderate antioxidant activity and negative health effects [10]. In view of the importance of antioxidants, demand for natural antioxidants with potential beneficial effects on human health is increasing [11, 12]. Thus far, plants have been the main source of natural antioxidants owing to their high antioxidant content [13, 14]. However, microbial species are known as an immense reservoir of pharmaceutically active compounds and has gained increasing attention in drug discovery [15, 16].

In the field of microbial drug discovery, actinobacteria have been greatly studied for their ability to make a wide range of novel and highly potent bioactive compounds; accounting for 45% of all the discovered bioactive metabolites [17, 18]. The dominant and best-studied genus of this phylum is *Streptomyces* which has a remarkable contribution to mankind since the golden era of drug discovery was initiated with the discovery of streptomycin from *Streptomyces griseus* [12, 19, 20]. At present, *Streptomyces* accounts for 70–80% of relevant bioactive metabolites produced by more than 500 species with diverse biological activities such as antibacterial, antifungal, antioxidant, anticancer, anti-inflammatory and anti-parasitic [21]. However, there are very limited studies on *Streptomyces* with respect to phenolic compounds as antioxidants. So, there is need to screen more streptomycetes with potent free radical scavenging activity. Rhizospheric *Streptomyces* have great importance in search of novel species and new bioactive compounds with diverse biological activities. Due to the presence of various complex interactions in the rhizosphere, microorganisms have coevolved with plants and show similar type of structure and function [22]. Various studies reported the potential of *Streptomyces* spp. from one such valuable region [23–25].

In the light of this, during our screening programme for isolation of rhizospheric *Streptomyces* spp. exhibiting different bioactivities, a potent streptomycete isolate indexed as TES17 was isolated from rhizospheric soil of tea (*Camellia sinensis* L.; family Theaceae) plant collected from Palampur (Himachal Pradesh, India). Tea is the most popular and widely consumed beverage in world second to water [26]. The numerous health benefits associated with tea consumption have been attributed to the free radical-scavenging capabilities of the most abundant compounds such as tea catechins (up to 30% of dry weight), quercetin and myricetin [27–29].

Keeping this in mind, the tea rhizospheric soil strain TES17 was evaluated for antioxidant activities using various in vitro free radical scavenging assays and identified using polyphasic approach. To further support, DNA damage protective activity of *Streptomyces* TES17 was assessed using an in vitro DNA nicking assay, and cytotoxicity of the extract was evaluated on lung cancer cell line. The chemical constituents of the extract responsible for antioxidant activity were determined through ultra high-pressure liquid chromatography (UPLC) analysis.

## Methods

### Sample collection and isolation of *S. cellulosae* strain TES17

In July 2013, strain TES17 was isolated from soil sample collected from rhizosphere of tea (*Camellia sinensis*) plant, from the designated site TES- PM (32° 6′ 37.9” N 76° 32′ 10.4″ E), situated in Palampur, Himachal Pradesh (India). The plant has been formally identified by Department of Botanical and Environmental Sciences, Guru Nanak Dev University, Amritsar. The isolation of TES17 was done using the protocol described by Sharma et al. [30]. The isolate was maintained on Starch Casein Nitrate Agar medium (SCNA; g/L): starch 10.0, casein 0.3, KNO_3_ 2.0, NaCl 2.0, K_2_HPO_4_ 2.0, MgSO_4_.7H_2_O 0.05, CaCO_3_ 0.02, FeSO_4_.7H_2_O 0.01 and agar 20.0) slants at 4 °C in the refrigerator and as spore suspensions in 20% *v/v* glycerol at − 70 °C in an ultra-low temperature freezer.

### Characterization and identification of isolate TES17 using polyphasic approach

#### Phenotypic and biochemical characterization

Various cultural and morphological properties such as sporulation, pigmentation of aerial and substrate mycelia and soluble pigmentation in medium which are highly characteristic and useful in the classification of the genus *Streptomyces* [31] were observed by inoculating the TES17 on SCNA and different ISP media (ISP-1, ISP-2, ISP-3, ISP-4, ISP-5, ISP-6 and ISP-7) as per methods prescribed in International *Streptomyces* Project [32]. Morphological properties of the isolate were observed by the light microscope (Olympus) at 100X, and spore chain and spore surface morphology was determined by scanning electron microscopy (Carl Zeiss model EVOLS 10). Physiological tests were performed by growing the strain on SCNA at different temperatures (20–50 °C), pH (5.0–12.0) and different NaCl concentrations (0–20% *w*/*v*). Assimilation of sugars as carbon sources (1%) was studied according to Shirling and Gottlieb [32]. D-glucose, arabinose, xylose, adonitol, rhamnose, cellobiose, lactose, malonate, raffinose and trehalose (1%) (HiMedia, India) were added to the basal medium after filter sterilization. Catalase production, citrate utilization, hydrolysis of urea, esculin, casein, gelatin and starch were performed using the method given by Cowan and Steel [33]. Indole production and Methyl red and Vogues Proskauer (MR-VP) tests were performed as recommended by Holding and Collee [34]. Nitrate reduction was investigated according to Lanyi [35].

#### Genomic characterization based on 16S rRNA gene sequencing

For 16S rRNA gene sequencing, the genomic DNA of the isolate was extracted following the method given by Marmur [36], and amplification was performed by polymerase chain reaction (PCR) using primers 27f (5’-AGAGTTTGATCCTGGCTCAG-3′) and 1492r (5’-AGAAAGGAGGTGATCCAGGC-3′). The amplified PCR product was purified using QIA quick gel extraction kit (Qiagen, Germany). The purified PCR product got sequenced from Institute of Microbial Technology (IMTECH), Chandigarh, India. Identification of phylogenetic neighbors and calculation of pair wise 16S rRNA gene sequence similarities were achieved using the EzTaxon server (http://www.ezbiocloud.net) [37]. The almost complete sequence (1445 bp) was aligned using Clustal W Program. Phylogenetic trees were constructed according to the neighbor joining, maximum-parsimony and maximum likelihood algorithms using bootstrap values based on 1000 replications with the MEGA 6.0 software [38, 39].

### Extract preparation of strain TES17

The fermentation process was carried out by inoculating the production medium with seed culture. To prepare seed culture, the seven-day old actinobacterial plate was inoculated in 100 mL of SCN broth. After 48 h, inoculum (2%) was aseptically transferred to 250 mL of Erlenmeyer flask containing 50 mL of the production medium same as seed medium and incubated for four days at 28 °C under shaking at 180 rpm. After fermentation, the broth was centrifuged at 10,000X g for 20 min at 4 °C and the supernatant was separated. For the extraction of bioactive compound/s, the supernatant was extracted with ethyl acetate twice in the ratio of 1:1 (supernatant: solvent) using separating funnel. The organic phase was then concentrated to dryness using rotavapor (BUCHI) at 40 °C. The crude extract obtained was dissolved in methanol prior to further analysis.

### Antioxidant potential of *S. cellulosae* strain TES17 extract

#### DPPH radical scavenging assay

Antioxidant activity of the TES17 extract was examined using DPPH (2,2-diphenyl-1-picrylhydrazyl) assay according to the method given by Kumar et al. [10] with minor modifications. Varying concentrations (0.5–5 mg/mL) of TES17 extract were mixed with freshly prepared 0.002% (*w*/*v*) DPPH (Sigma-Aldrich) in methanol. The reaction mixture was incubated in the dark at room temperature for 30 min prior to the measurement of absorbance at 517 nm with a microplate reader. Vitamin C was used as the positive control. The percentage DPPH radical inhibition was computed according to the following formula:

$$ \mathrm{DPPH}\ \mathrm{Scavenging}\ \mathrm{Activity}\ \left(\%\right)=\left[\left(\mathrm{Ao}\hbox{--} \mathrm{Ae}\right)/\mathrm{Ao}\right]\times 100 $$

Where Ao is the absorbance of the control and Ae is the absorbance of the metabolite/standard.

#### ABTS radical scavenging activity

The 2,2′- azino-bis (3-ethylbenzothiazoline-6-sulfonic acid) (ABTS) radical scavenging assay was conducted according to the method described by Re et al. [40] with small modifications. The ABTS free radical solution (190 μL) was reacted with the series of different concentrations (10 μL, 0.5–5 mg/mL) of the TES17 extract in 96-well microtitre plate. The ABTS free radical solution was prepared via reacting ABTS stock solution (7 mM) with potassium persulphate (2.45 mM) in dark for 24 h. The prepared ABTS radical solution was diluted to an absorbance of 0.7 ± 0.2 prior to performing the assay. The reaction was left in the dark at room temperature for 3 min before taking the absorbance at 743 nm. Ascorbic acid was used as positive control. The percentage of ABTS radical inhibition activity was indicated by the decrease in the absorbance of color produced by ABTS radical solution and was determined using the formula:

$$ \mathrm{ABTS}\ \mathrm{Scavenging}\ \mathrm{Activity}\ \left(\%\right)=\left[\left(\mathrm{Ao}\hbox{--} \mathrm{Ae}\right)/\mathrm{Ao}\right]\times 100 $$

Where Ao is the absorbance of the control and Ae is the absorbance of the metabolite/standard.

#### Superoxide anion scavenging assay

This assay was proposed by Nishikimi et al. [41] and used for measuring the superoxide anion scavenging potential. The process of generation of superoxide anions was non-enzymatic in a PMS-NADH system constituted of phenazine methosulphate and reduced nicotinamide adenine dinucleotide. Blue colored formazan dye was formed by reduction of nitro blue tetrazolium. In this method, TES17 extract (0.5–5 mg/mL) was mixed with 156 μM NADH (1 mL), 60 μM NBT (1 mL) and 468 μM phenazine methosulphate (1 mL) in phosphate buffer (pH = 8.3). PMS was added for initiation of reaction mixture followed by its incubation at 25 °C for 10 min. Gallic acid was used as positive control. The absorbance was read at 560 nm and the percentage inhibition was calculated using the formula:

$$ \mathrm{Superoxide}\ \mathrm{Anion}\ \mathrm{Scavenging}\ \mathrm{Activity}\ \left(\%\right)=\left[\left(\mathrm{Ao}\hbox{--} \mathrm{Ae}\right)/\mathrm{Ao}\right]\times 100 $$

Where Ao is the absorbance of the control and Ae is the absorbance of the metabolite/standard.

#### Phosphomolybdenum assay

The molybdate ion reducing ability of the extracts was assessed according to the method proposed by Prieto et al. [42] with small changes. In short, TES17 extract (5 mg/mL) was mixed with 900 μL of reagent solution which was comprised of 0.6 M sulphuric acid, 28 mM sodium phosphate and 4 mM ammonium molybdate. This mixture was kept for incubation for 90 min at 95 °C and followed by cooling at room temperature. The absorbance was read at 695 nm. The standard curve was plotted with 20–200 μg/mL concentrations of ascorbic acid. The total antioxidant capability was calculated from the standard curve and expressed as mg Ascorbic Acid Equivalents (AAE)/ 100 mg dry weight of extracts.

#### Reducing power assay

The total reducing power of TES17 extract was measured as described by Oktay et al. [43]. The assay measures the formation of a blue colored complex with potassium ferricyanide (K_3_Fe(CN)_6_), trichloroacetic acid and ferric chloride (FeCl_3_). Briefly, TES17 extract (5 mg/mL) was reacted with phosphate buffer (2.5 mL, 0.2 M, pH 6.6) and potassium ferricyanide (2.5 mL, 1% *w*/*v*). The mixture was incubated for 20 min at 50 °C and then added a small portion of trichloroacetic acid (2.5 mL, 10% *w/v*). The resulting mixture was centrifuged at 10000X g for 10 min. The collected supernatant (2.5 mL) was mixed with distilled water (2.5 mL) prior to the addition of FeCl_3_ (500 μL, 0.1% *w/v*). The absorbance was taken at 700 nm spectrophotometrically and compared with Vitamin C which was used as positive control. The reducing capability was calculated from the standard curve and expressed as mg Ascorbic Acid Equivalents (AAE) / 100 mg dry weight of extracts.

#### Lipid peroxidation assay

The method proposed by Dasgupta and De [44] with slight modifications was followed to estimate the amount of malondialdehyde (MDA). The protective ability of extract was demonstrated by mixing the different concentrations of TES17 extract (0.5–5 mg/mL) with 10% egg yolk (500 μL). For initiation of lipid peroxidation, 50 μL of 7 mM FeSO_4_ was added and incubated at 37 °C for 30 min. The process of lipid peroxidation was checked by the formation of thiobarbituric acid reactive substances (TBARS). TBARS were determined by adding ice-cold 20% trichloroacetic acid (TCA; 50 μL), 0.8% (*w*/*v*) TBA (1.5 mL) and 20% acetic acid (1.5 mL, pH 3.5) to the reaction mixture. The reaction mixture was heated at 100 °C for 60 min. After that, the reaction mixture was cooled and centrifuged at 3000X g for 10 min and the product was measured by spectrophotometer at 532 nm. The percentage inhibition was calculated with the formula:

$$ \mathrm{Inhibition}\ \mathrm{of}\ \mathrm{the}\ \mathrm{lipid}\ \mathrm{peroxidation}\ \left(\%\right)=\left[\left(\mathrm{Ao}\hbox{--} \mathrm{Ae}\right)/\mathrm{Ao}\right]\times 100 $$

Where Ao is the absorbance of the control and Ae is the absorbance of the metabolite/standard.

### Estimation of Total phenolic content (TPC) with Folin-Ciocalteu’s reagent method

The total phenolic content in the TES17 extract was estimated using Folin-ciocalteau method in 96-well microtitre plate according to Yu et al. [45] with slight modifications. 10 μL of the TES17 extract (5 mg/mL) was mixed with 100 μL of Folin Ciocalteau Reagent. After 5 min of incubation at room temperature, 80 μL of 7.5% sodium carbonate (Na_2_CO_3_) was added to each well followed by 30 min incubation at room temperature in dark. The absorbance was measured with a microplate reader at 750 nm. The calibration curve of gallic acid (used as a standard) was plotted using the calibration curve and the amount of TPC was estimated as mg of Gallic Acid Equivalents (GAE)/100 mg of the extract.

### Estimation of Total flavonoid content (TFC) based on Aluminium-Flavanoid complexes formation method

The TFC in TES17 extract was quantified using the 96 well plate method described by Tan et al. [3]. In this, 100 μL of distilled water was added into each of the well, followed by 25 μL of the test sample (5 mg/mL) and 10 μL of 5% sodium nitrite (NaNO_2_). After 5 min of incubation, 15 μL of 10% aluminum chloride (AlCl_3_) was added and the reaction mixture was allowed to stand for 6 min before the addition of 50 μL of 1 M NaOH. Next, 50 μL of distilled water was added to each well of the microtiter plate and mixed well. The absorbance was recorded immediately at 510 nm using microplate reader. Catechin was used as positive control and used for plotting the calibration curve. Total flavonoid content (TFC) was measured as mg of catechin equivalent/100 mg dried weight of the extract.

### DNA nicking assay

This assay was performed according to the method given by Lee et al. [46]. This method was used to evaluate the potential of TES17 extract to protect super coiled pBR 322 plasmid from destroying effects of hydroxyl radicals produced by Fenton’s reagent. The reaction mixture constituted of 1 μL plasmid DNA (50 μg/100 μL), 10 μL Fenton’s reagent (30 mM H_2_O_2_, 50 μM ascorbic acid, and 80 μM FeCl_3_) and different concentrations of extract (200 μg/mL, 400 μg/mL, 600 μg/mL, 800 μg/mL and 1000 μg/mL) and distilled water to make the final volume up to 20 μL. An equal volume of distilled water was added in place of Fenton’s reagent in the negative control. It was followed by incubation at 37 °C for 30 min. The analysis of DNA was done on 1% agarose gel electrophoresis. The positive control used was rutin. Densitometric analysis was done to examine the DNA damage quantitatively with the help of GelQuant.NET (Version 1.8.2; BiochemLabSolutions.com) software. The percentage of different forms of DNA i.e. supercoiled (Form I), single standed nicked (Form II) and double stranded nicked and linear (Form III) was calculated.

### In vitro cytotoxic assay

#### Cell culture maintenance and treatment

HEK-293 T (Human Embryonic Kidney normal cell line) and A549 (adenocarcinoma lung cancer cell line) were obtained from National Centre for Cell Science (NCCS), Pune (India) and were maintained in complete growth medium Roswell Park Memorial Institute (RPMI) 1640 supplemented with 10% Fetal Bovine Serum along with antibiotics (100 μg/mL streptomycin and 100 Units/mL penicillin). The cell lines were maintained at 37 °C in an environment of 90% relative humidity and 5% CO_2_. To check the degree of confluency and absence of bacterial and fungal contamination, cultures were viewed using trypan blue dye in phase contrast microscope.

#### Measurement of cell viability using 3-(4,5-Dimethylthiazol-2yl)-2,5-diphenyl tetrazolium bromide (MTT) assay

The cytotoxicity of the TES17 extract was examined using MTT assay following the protocol previously described by Mossman [47]. It is a colorimetric assay that measures the reduction of yellow colored dye MTT by mitochondrial enzyme viz., succinate dehydrogenase. MTT enters mitochondria of the cell where it is reduced to an insoluble purple colored formazan product. The cells are then solubilized with an organic solvent like isopropanol or DMSO and the released solubilized formazan product is measured spectrophotometrically. Since the reduction of MTT can occur only in living cells, this assay is regarded as a measure of the viability of the cells. Cells (5000 cells/well) were seeded in the 96 well microtiter plate and allowed to adhere overnight. Varying concentrations of TES17 extract (30, 50, 100, 250 and 500 μg/mL) were then added to each well and further incubated for 42 h. After that, 100 μL of 0.5 mg/mL of MTT dye (Sigma-Aldrich) was added to each well and the plates were incubated at 37 °C in humidified environment with 5% CO_2_ for 4 h. Then, the complete medium was aspirated from the wells and blue formazan crystals formed by MTT reaction were dissolved in 100 μL of DMSO. The color was measured at 595 nm using a microplate reader. The proliferation of cells under treatment was assessed according to following formula:

$$ \mathrm{Cell}\ \mathrm{viability}\ \left(\%\right)=\left[\mathrm{Ae}/\mathrm{Ao}\right]\times 100 $$

Where Ao is the absorbance of the untreated cells (only medium) and Ae is the absorbance of the treated cells (with extract).

### Determination of phenolic compounds: Ultra performance liquid chromatography (UPLC) analysis

The UPLC analysis was performed on Nexera UHPLC (Shimadzu) system. The system was equipped with LC-30 AD quaternary gradient pump, SPD-M20 A diode array detector (DAD), CBM-20 A Communication Bus Module, CTO-10 AS VP column oven, DGU-10 A_5_ prominence degasser, SIL-30 AC Nexera auto sampler, C-18 column of dimensions 150 × 4.6 × 5 μm particle size, and combination of water and 70% methanol as mobile phase at flow rate of 1 mL/min were used for analysis. The column temperature was maintained at 27 °C and the run time of the sample was 26 min. The constituents were identified by comparison of their retention time with standards available.

### Statistical analysis

The antioxidant assays were performed in triplicate. Results were expressed in mean ± standard deviation (SD). SPSS statistical analysis software (Version 20.0; IBM SPSS) was used to analyze the data. Regression studies were carried out along with the analysis of multiple comparisons by using one-way analysis of variance (ANOVA) with post hoc Tukey’s test. Statistical significance was considered at *p* ≤ 0.05. The calculation of IC_50_ value i.e. the concentration of extract in mg/mL used for scavenging 50% free radicals was determined from the regression equation. Correlation analysis was also done to determine the relationship between the phenolic content, total flavonoid content and the antioxidant capacity of the extract using SPSS.

## Results

### Phenotypic analysis of strain *S. cellulosae* TES17

**Fig. 1:**
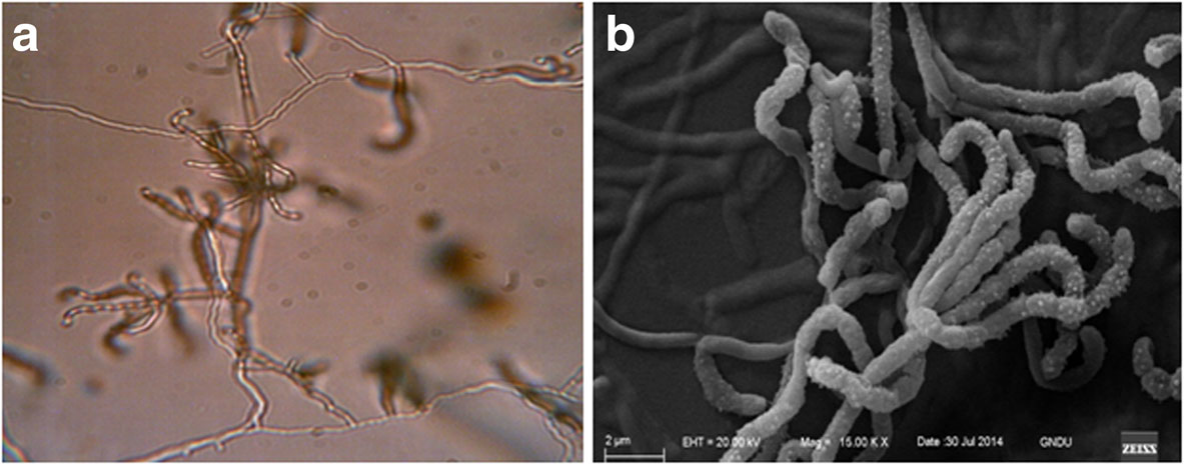
(**a**) Light micrograph at 100X showing flexous spore chains on aerial mycelium of *Streptomyces* TES17 (**b**) Electron microscopic view of spores showing rough surface

The strain TES17 showed gray sporulation with cream color aerial mycelium, while the substrate mycelium was of dark brown color on SCNA medium. In light microscope (100X), flexuous sporophores with short chains were observed and placed in Rectus-Flexibilis (RF) group of *Streptomyces* (Fig. 1a). Micromorphological studies by SEM revealed the branched spore chains, bearing 7–12 cylindrical spores (1.5–2.0 μm X 1.5 μm) having a rough surface (Fig. 1b). Culture characteristics of the strain are described in Table 1.

**Table 1:** Cultural characteristics of strain TES17 on various media

Medium	Sporulation	S.M.^a^	A.M.^b^	Growth	Pigmentation
ISP1	–	Off white	–	Poor	–
ISP2	Dark gray	Dark brown	Light brown	Good	–
ISP3	Light gray	Dark brown	Light brown	Good	–
ISP4	Dark gray	Black	Cream	Good	–
ISP5	Gray	Pale yellow	Cream	Good	–
ISP6	–	Light brown	Light brown	Good	–
ISP7	Off white	Cream	Cream	Good	–

^a^Substrate mycelium, ^b^Aerial mycelium, (−) = negative

The culture grew well on all the ISP media except tryptone yeast agar (ISP 1). Pigmentation was not observed in any of the media. Strain TES17 did not produce melanin pigment on tyrosine agar (ISP 7). These cultural characteristics of strain TES17 were in line with the genus *Streptomyces*. The isolate could grow between 18 °C to 50 °C (optimum at 28 °C), pH 5.0 to 12.0 (optimum at pH 7.0). It could tolerate NaCl concentration up to 2.5%. The strain TES17 hydrolyzed starch, lipid, gelatin, and esculin but gave negative results for both indole production and MRVP test. In the enzymatic activity, it could produce industrially important enzymes such as cellulase, urease, β-galactosidase and citrase by degrading their respective substrates cellulose, urea, ortho-Nitrophenyl-β-galactoside (ONPG) and citrate. However, the strain could neither reduce nitrate nor produce H_2_S. Strain TES17 was able to utilize glucose, arabinose and malonate as sole carbon source, showed weak growth on medium containing xylose, rhamnose and cellobiose, but didn’t utilize the raffinose and trehalose (Table 2).

**Table 2:** Morphological, physiological and biochemical characteristics of *Streptomyces* TES17

Properties	Result
Morphological characteristics	
Sporophore morphology	Rectus-flexibilis (RF)
Aerial mycelium colour	Cream
Substrate mycelium colour	Dark brown
Spore surface	Rough
Physiological characteristics	
Production of melanoid pigment	–
Temperature range for growth	18 °C to 50 °C
Optimum temperature for growth	28 °C
pH range for growth	5 to 12
Optimum pH for growth	7.0
NaCl tolerance	Up to 2.5%
Biochemical characteristics	
Gelatin hydrolysis	+
Starch hydrolysis	+
Lipid hydrolysis	+
Cellulase production	+
Citrate utilization	+
MRVP	–
Indole production	–
Urease production	+
Nitrate reduction	–
Esculin hydrolysis	+
H_2_S production	–
ONPG^a^ hydrolysis	+
Catalase test	+
Oxidase test	+
Utilization of:	
Glucose	+
Arabinose	+
Xylose	+/−
Adonitol	–
Rhamnose	+/−
Cellobiose	+/−
Lactose	–
Malonate	+
Raffinose	–
Trehalose	–

Positive = (+), negative = (−), doubtful = (+/−), ^a^ortho-Nitrophenyl-β-galactoside

### Phylogenetic and genomic analyses based on 16S rRNA sequencing

**Fig. 2:**
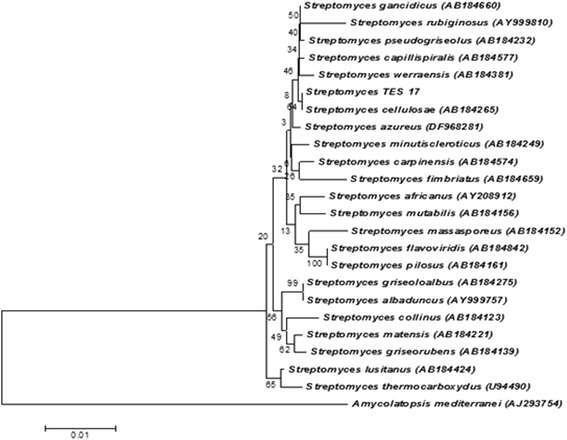
Neighbor-joining algorithm based on nearly complete 16S rRNA gene sequences showing the position of *Streptomyces* TES17 amongst its phylogenetic neighbors. Bootstrap values (expressed as percentages of 1000 replications) are shown at the nodes. *Amycolatopsis mediterranei* (AJ293754.1) was used as an out group. GenBank accession numbers are given in parentheses

The almost complete 16S rRNA gene sequence (1445 bp) of strain TES17 was determined and deposited in GenBank under accession no. KY511722. The sequence then compared with nucleotide sequences of other closely related taxa from the EzTaxon database. It showed 100% sequence similarity with *Streptomyces cellulosae* (AB184265). Further confirmation was done by constructing the phylogenetic trees using neighbor-joining, maximum-parsimony and maximum likelihood algorithms (Fig. 2, Additional files 1 and 2). High boot strap value of 64 in neighbor-joining algorithm further validated that the strain TES17 belongs to *S. cellulosae*. Therefore, on the basis of phylogenetic analysis, the isolate TES17 was designated as *S. cellulosae* strain TES17.

### Antioxidant activity of *Streptomyces* TES17

Further, antioxidant potential of *S. cellulosae* strain TES17 was investigated based on various in vitro antioxidant assays including the DPPH radical scavenging, ABTS radical scavenging, superoxide radical scavenging assays, total reducing power assay and total antioxidant capacity (TAC) was determined by phosphomolybdenum assay. The results of these assays based upon percentage inhibition of free radicals are tabulated in Table 3. In this study, extract of TES17 exhibited significant dose dependent DPPH radical scavenging activity measured from 12.66 ± 1.17 to 78.47 ± 0.23% (*p* ≤ 0.05) at concentrations ranging from 0.5 to 5 mg/mL, indicating the hydrogen donating ability of the TES17 extract to DPPH radicals. IC_50_ value i.e. 50% inhibition of DPPH radicals was achieved at concentration of 2.48 mg/mL. ABTS assay also revealed the potential of TES17 extract by scavenging the ABTS radical cations measuring from 67.73 ± 2.76 to 91.08 ± 0.98% (*p* ≤ 0.05) at concentrations ranging from 0.5 to 5 mg/mL. The TES17 extract was also tested for its scavenging capability against superoxide radicals (O_2_^•-^). In this assay, conversion of highly water soluble Nitroblue Tetrazolium (NBT) salt into water soluble NBT di-formazan dye takes place via reduction of superoxide radical. The rate of the reduction with O_2_^•-^ is linearly related to the activity of xanthine oxidase (XO) which is inhibited by superoxide dismutase (SOD). The results showed significant activity measuring from 10.37 ± 2.09 to 82.08 ± 0.93% (*p* ≤ 0.05) at different doses ranging from 0.5 to 5 mg/mL. Extract showed 50% inhibition i.e. IC_50_ at 2.41 mg/mL.

**Table 3:** The antioxidant activities demonstrated by TES17 extract in different antioxidant assays

Concentration of TES17 extract (mg/mL)	Antioxidant activities		
	DPPH radical scavenging activity (%)	ABTS radical scavenging activity (%)	Superoxide anion scavenging assay (%)
0.5	12.66 ± 1.17*	67.73 ± 2.76*	10.37 ± 2.09*
1	34.55 ± 0.14*	72.39 ± 1.27*	27.50 ± 0.77*
2	48.88 ± 2.12*	76.25 ± 1.65*	52.88 ± 1.04*
3	64.91 ± 1.58*	82.65 ± 1.73*	68.02 ± 0.72*
4	69.86 ± 2.48*	87.40 ± 1.21*	75.65 ± 0.94*
5	78.47 ± 0.23*	91.08 ± 0.98*	82.08 ± 0.93*

*Statistically significant (*p* ≤ 0.05) when compared to control (without extract)

Total antioxidant activity and total reducing power was expressed as mg Ascorbic Acid Equivalents (AAE)/100 mg dry weight of extracts. The higher antioxidant capacity was indicated by the high absorbance at a particular wavelength. Molybdate ion and ferric ion reduction potential were found to be 76.93 ± 0.76 and 231.96 ± 0.51 mg AAE/100 mg dry weight of TES17 extract, respectively.

### Lipid peroxidation assay

TES17 extract was also tested for in vivo effects on oxidative damage induced by free radicals on polyunsaturated fatty acids which results in the generation of mutagenic and toxic products, especially MDA. TES17 extract showed inhibition to lipid peroxidation as demonstrated by significant decrease in the relative percentage of MDA level (from 100 to 32.88%). The percentage inhibition at all the tested concentrations (ranging from 0.5 to 5 mg/mL) varied from 4.44 ± 0.34 to 67.18 ± 1.9% and shown as statistically significant at *p* ≤ 0.05 (Table 4).

**Table 4:** Inhibition of lipid peroxidation and effect of TES17 extract on the MDA level in egg homogenate

Concentration of TES17 extract (mg/mL)	Percentage inhibition	Relative percentage of MDA
0.5	4.44 ± 0.34*	95.61*
1	8.78 ± 0.68*	91.21*
2	15.64 ± 2.36*	84.39*
3	27.57 ± 1.11*	72.42*
4	52.48 ± 0.11*	47.58*
5	67.18 ± 1.9*	32.88*

All data are presented as the mean ± SD (*n* = 3). *Denotes *p* ≤ 0.05 between control sample (without extract) and TES17 extract treated sample

### Phenolic and flavonoid contents of TES17 extract

In the calculation of TPC of the extract, the phenolic compounds in the extract undergo reaction with phosphomolybdic acid in the presence of Folin-Ciocalteau reagent and lead to the production of a blue coloured complex in alkaline medium which is directly correlated with high antioxidant activity. The TPC of TES17 extract was expressed as Gallic Acid Equivalents/100 mg of dry weight of extract which was accounted as 55 mg GAE/100 mg of extract. Along with phenolics, flavonoids also are the major part of secondary metabolites which were quantified using colorimetric assay. In this, aluminum chloride forms acid stable complexes with the C-4 keto group or either with the C-3 or C-5 hydroxyl group of flavones and flavonols. In addition, aluminum chloride forms acid-labile complexes with the orthodihydroxyl groups in the A- or B-ring of flavonoids, absorbed at 510 nm. Total flavonoid content of TES17 extract was measured as 11.17 ± 4.05 mg rutin/100 mg of extract.

To assess the relationship between the antioxidant capacity using different antioxidant assays, TPC and TFC of TES17 extract, correlation analysis was performed. The perason’s correlation coefficients between these variables are presented in Table 5. The analysis clearly indicated that the highest correlation was found between ABTS radical scavenging activity, TPC and TFC of the extract with *r* = 0.995 and 0.985, respectively (*p* ≤ 0.01). The analysis also suggested that the antioxidant activity was largely contributed by the phenolic compounds, especially flavonoids present in the TES17 extract.

**Table 5:** Pearson’s correlation coefficients between TPC, TFC and antioxidant activities of TES17 extract

Antioxidant activities	Total Phenolic Content	Total Flavonoid Content
DPPH radical scavenging activity	*r* = 0.978**	*r* = 0.945**
ABTS radical scavenging activity	*r* = 0.995**	*r* = 0.985**
Superoxide anion scavenging activity	*r* = 0.974**	*r* = 0.933**
Lipid peroxidation activity	*r* = 0.964**	*r* = 0.986**

**Correlation is significant at the *p* ≤ 0.001 level

### DNA protective effect against oxidative damage induced by H_2_O_2_ using DNA nicking assay

**Fig. 3:**
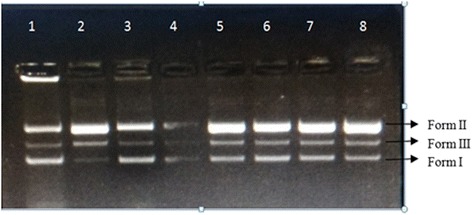
DNA Nicking assay revealing protective potential of TES17 extract against free radicals generated by Fenton’s reagent. Lane 1: Negative control (only DNA); Lane 2: Fenton’s reagent; Lane 3: Positive control (rutin, 10 μg); Lane 4–8: Fenton’s reagent + 2, 4, 6, 8 and 10 μg of TES17 extract; Form I = Supercoiled; Form II = Linear; Form III = Single strand nicked DNA

The DNA nicking assay revealed that TES17 extract protected the supercoiled DNA pBR322 from the destructive effects of hydroxyl radicals generated by Fenton’s reagent. It was observed that supercoiled form of plasmid DNA (Form I; Lane 1) was degraded to single stranded and double stranded nicked and linear forms of DNA (Form II and III, respectively; Lane 2) due to hydroxyl radicals generated in Fenton’s reaction mixture. However, addition of TES17 extract at different concentrations (from 2 to 10 μg/well) to the reaction mixture minimized the hydroxyl radical mediated DNA damage i.e. conversion of supercoiled DNA (Form I) to the formation of single stranded nicked DNA (Form II) and double stranded nicked and linear DNA (Form III) as shown in lanes 4–8. Lane 3 shows the positive control rutin, which maintained the integrity of the DNA to the Form I (Fig. 3).

**Fig. 4:**
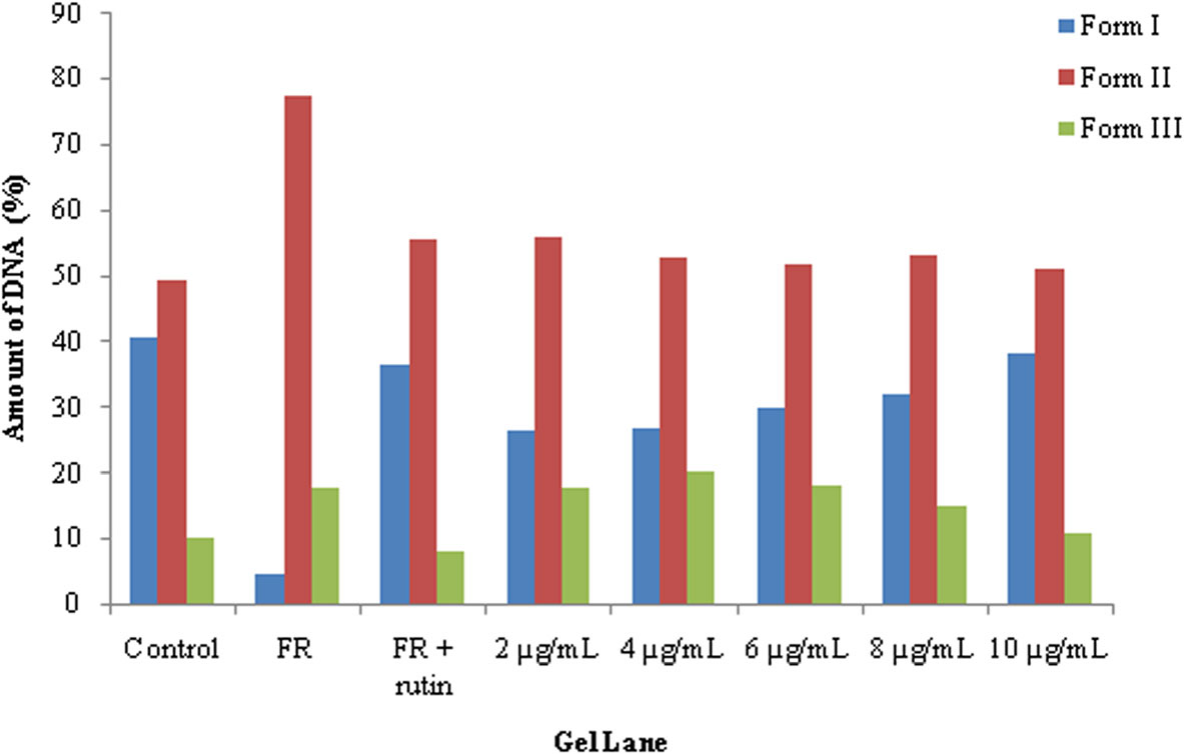
Densitometric analysis of DNA protective effects of TES17 extract in the presence of hydroxyl radicals generated in DNA nicking assay. Form I- supercoiled DNA, Form II- single stranded nicked DNA, Form III- double standed nicked and linear DNA

The densitometric analysis determined the percentage of DNA present in three forms. Figure 4 demonstrates that the amount of supercoiled DNA in the presence of TES17 extract and Fenton’s reagent was found to be 38.2% (10 μg), 31.82% (8 μg), 29.97% (6 μg), 26.89% (4 μg) and 26.38% (2 μg), indicating that the increasing concentration of extract protect the damaged DNA with higher intensity. The results were in comparison with the amount of supercoiled DNA present in positive control (rutin + Fenton’s reagent) with 36.54%. In the presence of Fenton’s reagent only, the amount of Form I was found to be 4.61% and simultaneously, Form II increased from 49.27% to 77.61% (Table 6).

**Table 6:** Densitometric analysis of different forms of DNA (in percentage) after treatment with varying concentrations of TES17 extract

Form	Control (only DNA)	^a^FR	Rutin (10 μg) + FR	Concentration of TES17 extract				
				2 μg	4 μg	6 μg	8 μg	10 μg
Form I	40.798	4.605	36.54	26.378	26.89	29.977	31.823	38.203
Form II	49.274	77.614	55.609	56.043	52.988	51.871	53.076	50.987
Form III	9.928	17.781	7.845	17.578	20.122	18.151	15.101	10.81

^a^FR = Fenton’s Reagent, Form I - supercoiled DNA, Form II - single stranded nicked DNA, Form III - double standed nicked and linear DNA

### Cytotoxic activity of *Streptomyces* TES17 extract

**Fig. 5:**
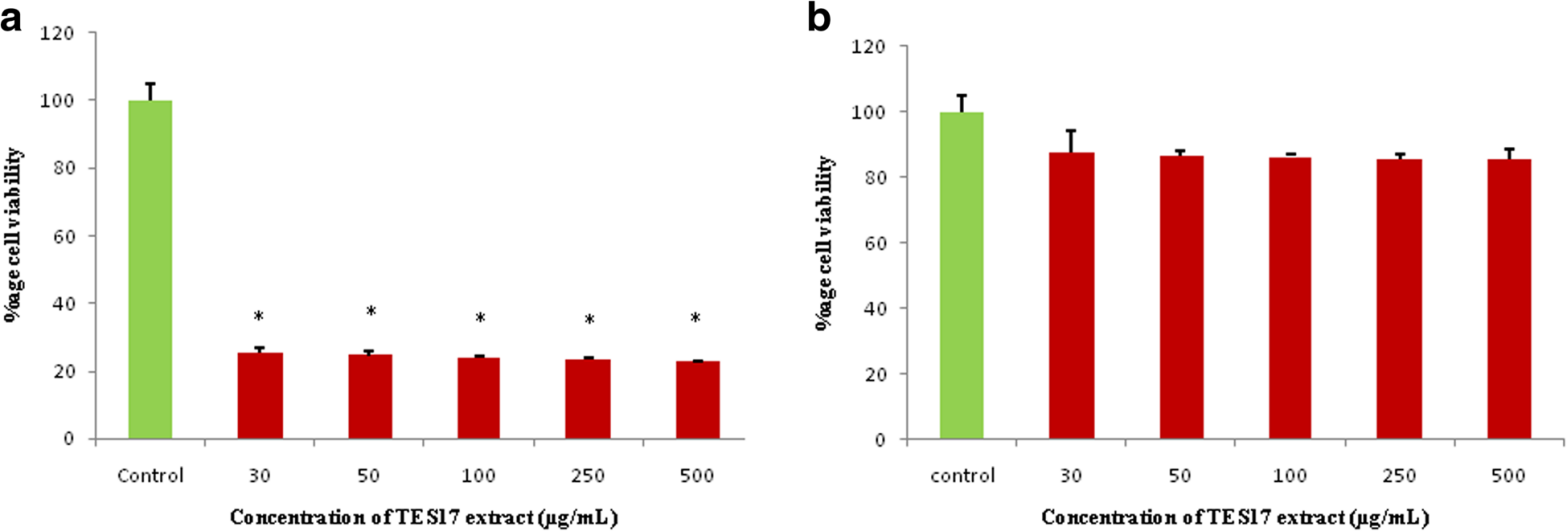
Effect of TES17 extract against (**a**) cancer cell line and (**b**) normal cell line. The viability of control cells (without TES17 extract) was defined as 100%. Data shown are mean ± SD (*n* = 3). ^*^ Denotes *p* ≤ 0.05 between control cells and treated cells

In addition, TES17 extract displayed cytotoxic activity against A549 lung cancer cell line with recording viability ranging from 25.3 ± 1.52 to 22.72 ± 0.34% (Fig. 5a). A dose response pattern, evaluated using different doses, indicated that the TES17 extract was highly effective even at the lowest tested concentration (30 μg/mL) with 74.7 ± 1.33% inhibition against cancer cell line and increased concentration didn’t show any effect. Furthermore, it was also observed that there was significant difference (*p* ≤ 0.05) between control and increasing concentration of the extract. As for the HEK-293 T (Human Embryonic Kidney normal cell line), TES17 extract displayed insignificant cytotoxic effect with 87.71 ± 6.66 to 85.41 ± 3.14% viability of the tested cells at different concentrations of the extract (Fig. 5b).

### Chemical profiling of *Streptomyces* TES17 extract using UPLC analysis

**Fig. 6:**
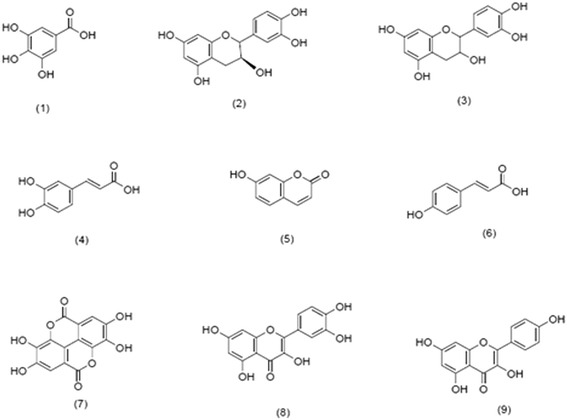
Chemical structures of phenolic compounds detected in TES17 extract

To determine the phenolic compounds that may be responsible for its antioxidant, DNA protective and cytotoxic properties, TES17 extract was subjected to UPLC analysis. The results of UPLC analysis revealed that it contained nine compounds (Table 7 and Additional file 3): Gallic acid (1), Catechin (2), Epicatechin (3), Caffeic acid (4), Umbelliferone (5), Coumaric acid (6), Ellagic acid (7), Quercetin (8) and Kaempferol (9) with chemical structures shown in Fig. 6. It was clearly analysed that phenolic acids and flavanoids were the main classes of phenolic compounds present in the extract.

**Table 7:** Phenolic compounds identified in TES17 extract

Sr. No.	Phenoic Compounds	Retention Time (min)	Concentration (mg/L)	Molecular Formula	Molecular Weight (MW)
1	Gallic acid	2.579	127.735	C_7_H_6_O_5_	170.12
2	Catechin	3.912	804.940	C_15_H_14_O_6_	290.27
3	Epicatechin	6.244	46.223	C_15_H_14_O_6_	290.27
4	Caffeic acid	6.951	39.985	C_9_H_8_O_4_	180.16
5	Umbelliferone	9.749	19.264	C_9_H_6_O_3_	162.14
6	Coumaric acid	10.290	0.652	C_9_H_8_O_3_	164.16
7	Ellagic acid	15.668	26.972	C_14_H_6_O_8_	302.19
8	Quercetin	16.426	12.485	C_15_H_10_O_7_	302.23
9	Kaempferol	17.554	8.943	C_15_H_10_O_6_	286.23

## Discussion

The ‘rhizosphere’, narrow and specific zone forms unique microhabitat from a bulk of the soil in terms of high nutrient availability, optimum pH and input of organic materials derived from root exudates [48]. These conditions favor the abundance of diverse microbial community in the rhizosphere. In well-studied rhizosphere, root exudates play an important role in enhanced biomass and activity of microorganisms [22, 49]. The complex interactions between plants and microorganisms in the rhizosphere for carbon sequestration, ecosystem functioning and nutrient cycling, lead to the production of novel bioactive metabolites [50]. Among microorganisms, *Streptomyces*, a largest known genus of actinobacteria which commonly inhabit rhizosphere soil, has been greatly explored as producer of a wide variety of unique metabolites with interesting biological activities [20, 51]. Therefore, it is an efficient approach to explore *Streptomyces* spp. from such environment for the discovery of novel bioactive compounds.

In the present study, *Streptomyces* strain TES17 isolated from rhizosphere of tea (*Camellia sinensis*) was characterized through the polyphasic approach which included morphological, physiological, biochemical molecular characterization, and phylogenetic analysis. It formed branched short spore chains having a rough surface as observed via SEM studies. The strain was capable to survive under unfeasible growth conditions because it could tolerate NaCl concentration of 2.5%, temperature up to 50 °C and pH 12.0. Furthermore, strain TES17 also could have the potential to gain attention in industrial sector due to the production of industrially important enzymes such as cellulase, amylase, and β-galactosidase. The type and quantity of secondary metabolites are greatly influenced by the availability of substrates given during the growth [52]. The results revealed that strain was capable to utilize different carbon sources such as glucose, arabinose, malonate, xylose, rhamnose, and cellobiose. Phylogenetic analysis based on 16S rRNA gene sequences showed that strain TES17 is closely related to *Streptomyces cellulosae* (AB184265) as they showed 100% sequence similarity and formed a distinct clade at the bootstrap value of 64% which was significantly higher than the threshold value of 50%. However, phenotypically the strain TES17 is different from reference strain *Streptomyces cellulosae* (AB184265) in terms of having rough spore surface [53]. Overall, the strain TES17 could belong to same species as *S. cellulosae* (AB184265) based on 16S rRNA sequencing. To the best of our knowledge, *S. cellulosae* strain TES17 has not been reported earlier for the antioxidant, DNA damage protective and anticancer activities.

Oxidative stress and other neurodegenerative diseases are associated with accumulation of free radicals or ROS [54, 55]. The process of oxidation may occur via different types of radicals which have different types of reaction mechanisms based on their interaction with surrounding molecules such as electron donation, reducing radicals, and electron acceptance [55, 56]. Hence, to assess the overall antioxidant potential of the extract, a total of six assays which elicit different mechanism of actions were performed. These assays demonstrated that the TES17 extract exhibited DPPH radical scavenging activity, ABTS radical scavenging activity, superoxide anion scavenging activity, reducing power of ferric ions and molybdate ions and ability to inhibit lipid peroxidation.

Simplest and robust methods to screen antioxidant activity are DPPH and ABTS assays. These assays involve stable free DPPH and ABTS• + radicals. Hydrogen donating potential of antioxidants is responsible for their effects on DPPH and ABTS radicals. The transfer of hydrogen atom or electron by the antioxidant molecule to the DPPH and ABTS radical leads to the decolorization of violet and bluish-green color, respectively [57]. Besides the hydrogen donating ability, TES17 extract also has the tendency to scavenge the superoxide anion radical (O_2_^•-^). Excessive generation of O_2_^•-^ (directly or indirectly) involves in the formation of other highly reactive hydrogen peroxides (H_2_O_2_), notorious hydroxyl radicals (OH^•^), peroxynitrite (ONOO^−^) or singlet oxygen species during the process of aging and pathological events which ultimately contribute to oxidative stress and carcinogenesis [58]. Thus, the increased production rate of O_2_^•-^ overwhelms the capacity of superoxide dismutase enzyme of an internal defense system. So, the need to control O_2_^•-^ production to prevent oxidative stress is of great importance [59].

Some of the assays including total reducing power, total antioxidant activity, total phenol content and total flavonoid content are the direct measure of presence of the phenolic compounds. Total reducing power of extract was determined by measuring the reduction of potassium ferricyanide (Fe^3+^) to potassium ferrocyanide (Fe^2+^) which further undergoes reaction with ferric chloride to produce ferric ferrous complex (which has intense blue color). The higher reducing potential of the extract was indicated by the increase in absorbance. Similarly, in molybdate ion reduction assay antioxidant compound reduces Mo (VI) to Mo (V) that results in the formation of green colored phosphate/ Mo (V) complex at acidic pH that determines the process of donation of electrons [42, 60].

The targets of ROS are mainly proteins, DNA, RNA molecules, sugars and lipids [61]. Lipids having many numbers of C=C bonds undergo easier oxidative deterioration resulting in the formation of monounsaturated (MUFA) and saturated fatty acids (SFA), more resistant to radicals than polyunsaturated fatty acids (PUFA). The process of lipid peroxidation is initiated by an attack towards a fatty acid’s side chain by a hydroxyl radical (OH^•^) produced by the interaction between hydrogen peroxide and iron metal ions present in Fenton reaction. This process abstracted a hydrogen atom from a methylene carbon which further undergoes molecular rearrangements and forms a peroxyl radical. The latter facilitates the production of the carcinogenic and mutagenic product MDA [55]. The reduction in MDA level by inhibiting the ferryl-perferryl complex and quenching the OH^•^ confirmed the significant role of TES17 extract in inhibiting lipid peroxidation [62].

To further support the antioxidant potential, this study demonstrated the DNA protective effect of TES17 extract using oxidative stress induced DNA damage model. In DNA nicking assay, O_2_^•-^ radical is produced by the autoxidation of Fe (II) which further generates OH^•^ by a rapid reaction of H_2_O_2_ in the presence of ascorbic acid as catalyst at pH 7.4. Ascorbate plays the role of reducing Fe (III) to Fe (II) making Fenton reaction to take place [63, 64]. A decrease in the single stranded or double stranded nicked (Form II) and linear forms of DNA (Form III), and simultaneous increase in the native supercoiled form (Form I) in the presence of extract confirmed protective effect of TES17 against ROS induced DNA damage.

Oxidative stress ultimately initiates cancer progression by various modifications in the biological molecules which eventually lead to increased mutation rate [65]. Various antioxidant assays revealed that the extract TES17 produced such bioactive compounds which could be further used as chemopreventive drugs to reduce cancer. An ideal chemotherapeutic drug should have high specificity i.e. able to differentiate cancer and normal cells. However, many of the drugs in use are still lacking in the drug specificity as they kill both cancer as well as normal cells [66, 67]. This study investigated the specificity of TES17 extract which revealed that the extract was highly toxic to A549 lung cancer cell line (25.3 ± 1.52 to 22.72 ± 0.34% viable cells) as compared to normal cell line (87.71 ± 6.66 to 85.41 ± 3.14% viable cells) at the different tested concentrations. These key findings could provide useful information for future development of *S. cellulosae* strain TES17 as the producer strain of anticancer drugs.

The correlation studies between the antioxidant assays, the total phenolics and favonoids in the TES17 extract suggested that phenolic compounds (both phenolic acids and flavonoids) made a significant contribution to the antioxidant potential of *Streptomyces* TES17. Further confirmation of phenolic compounds in the TES17 extract was done using HPLC analysis which is a powerful analytical tool and widely used for the detection of phenolic compounds based on the particular retention time [68]. Phenolic acids are derivatives of hydroxybenzoic acids and hydroxycinnamic acids such as gallic acid, caffeic acid and coumaric acid [69]. Second class of phenolic compounds are flavonoids which are mainly characterized as containing two phenolic rings (Ring A and Ring B) linked by another oxygenated heterocycle which is three carbon bridge (Ring C), forming a common diphenylpropane (C6-C3-C6) skeleton structure. Based upon the saturation level of the C ring, flavonoids are classified into different subclasses *viz.* flavonols, flavanols, anthocyanins, isoflavonoids, flavanones and flavones. Individual compounds within a subclass differ in the substitution pattern of hydroxyl groups present in the A and B rings that influence the free radical-scavenging properties of the phenolic compounds [70]. Some of the most important flavonoids are catechin, rutin, quercetin and kaempferol. Both phenolic acids and other flavonoids act as antioxidants by chelating the ions and scavenging he free radicals particularly, superoxide (O_2_^●-^), peroxyl and hydroxyl radicals (OH^●^) and hence inhibit both DNA damage and lipid peroxidation, which can cause membrane damage.

UPLC analysis of extract of *S. cellulosae* strain TES17, isolated from tea rhizosphere, revealed that among nine phenolic compounds [*viz*. catechin, epicatechin, quercetin and kaempferol (flavonoids), gallic acid, caffeic acid and coumaric acid (phenolic acids) and umbelliferone (coumarine)], catechins were the principal phenolic compounds. Catechins are reported to be the main phenolic compounds responsible for antioxidant activity of the tea. The principal catechins present in tea leaves are epigallocatechin gallate (EGCG), epigallocatechin (EGC), epicatechin gallate (ECG), gallocatechin (GC), epicatechin (EC), and catechin [28, 71]. These results suggest that there could be gene transfer between tea plant and rhizospheric microflora which further elaborate the potential of *Streptomyces* TES17 in search of phenolic compounds with interesting bioactivities.

## Conclusions

The present study showed the true antioxidant potential of *S. cellulosae* strain TES17 as it was able to scavenge several free radicals including DPPH, ABTS and superoxide anions; inhibit lipid peroxidation and protect DNA from the damage caused by oxidative stress. The biopharmaceutical importance of TES17 extract was further established by the cytotoxic activity against cancer cell line with high specificity. UPLC analysis demonstrated that the catechins were the major phenolic compounds in the extract responsible for various bioactivities. Therefore, the findings of the present study indicate that *S. cellulosae* strain TES17 holds promise for large scale production of free radical scavenging agents which can be developed as chemotherapeutic drugs used in the intervention of oxidative stress-mediated diseases.

## Additional files

Additional file 1: Maximum-parsimony phylogenetic tree based on 16S rRNA gene sequences of *Streptomyces* strains showing the position of isolate *Streptomyces* TES17. Bootstrap values (expressed as percentages of 1000 replications) are shown at the nodes. (DOCX 16 kb)

Additional file 2: Maximum-likelihood phylogenetic tree based on 16S rRNA gene sequences of *Streptomyces* strains showing the position of isolate *Streptomyces* TES17. Bootstrap values (expressed as percentages of 1000 replications) are shown at the nodes. (DOCX 15 kb)

Additional file 3: UPLC analyses of TES17 extract showing nine different phenolic compounds based on particular retention time. (DOCX 167 kb)

## Abbreviations

ABTS: 2,2′- azino-bis (3-ethylbenzothiazoline-6-sulfonic acid)
DPPH: 2,2-diphenyl-1-picrylhydrazyl
MTT: 3-(4,5-Dimethylthiazol-2yl)-2,5-Diphenyl Tetrazolium Bromide
ROS: Reactive oxygen species
SCNA: Starch casein nitrate agar medium
TFC: Total flavonoid content
TPC: Total phenolic content
UPLC: Ultra high pressure liquid chromatography
