# Supplementary material for: Phenolic compounds as antioxidants and chemopreventive drugs from Streptomyces cellulosae strain TES17 isolated from rhizosphere of Camellia sinensis
Source: BMC Complement Altern Med. 2018 Mar 9;18:82. doi: 10.1186/s12906-018-2154-4 (PMC5845325; doi:10.1186/s12906-018-2154-4)
Supplement: Supplementary file 1 — Maximum-parsimony phylogenetic tree based on 16S rRNA gene sequences of Streptomyces strains showing the position of isolate Streptomyces TES17. Bootstrap values (expressed as percentages of 1000 replications) are shown at the nodes. (DOCX 16 kb) [file 12906_2018_2154_MOESM1_ESM.docx]

**Phenolic compounds as antioxidants and chemopreventive drugs from *Streptomyces* *cellulosae* strain TES17 isolated from rhizosphere of *Camellia sinensis***

Riveka Rani^1^, Saroj Arora^2^, Jeevanjot Kaur^2^, Rajesh Kumari Manhas^1*^

^1^ Department of Microbiology, Guru Nanak Dev University, Amritsar, India, ^2 1^Department of Botanical and Environmental Sciences, Guru Nanak Dev University, Amritsar, India

**Additional File 1:** Maximum-parsimony phylogenetic tree based on 16S rRNA gene sequences of *Streptomyces* strains showing the position of isolate *Streptomyces* TES17. Bootstrap values (expressed as percentages of 1000 replications) are shown at the nodes.
