# Supplementary material for: Phenolic compounds as antioxidants and chemopreventive drugs from Streptomyces cellulosae strain TES17 isolated from rhizosphere of Camellia sinensis
Source: BMC Complement Altern Med. 2018 Mar 9;18:82. doi: 10.1186/s12906-018-2154-4 (PMC5845325; doi:10.1186/s12906-018-2154-4)
Supplement: Supplementary file 3 — UPLC analyses of TES17 extract showing nine different phenolic compounds based on particular retention time. (DOCX 167 kb) [file 12906_2018_2154_MOESM3_ESM.docx]

**Phenolic compounds as antioxidants and chemopreventive drugs from *Streptomyces* *cellulosae* strain TES17 isolated from rhizosphere of *Camellia sinensis***

Riveka Rani^1^, Saroj Arora^2^, Jeevanjot Kaur^2^, Rajesh Kumari Manhas^1*^

^1^ Department of Microbiology, Guru Nanak Dev University, Amritsar, India, ^2 1^Department of Botanical and Environmental Sciences, Guru Nanak Dev University, Amritsar, India


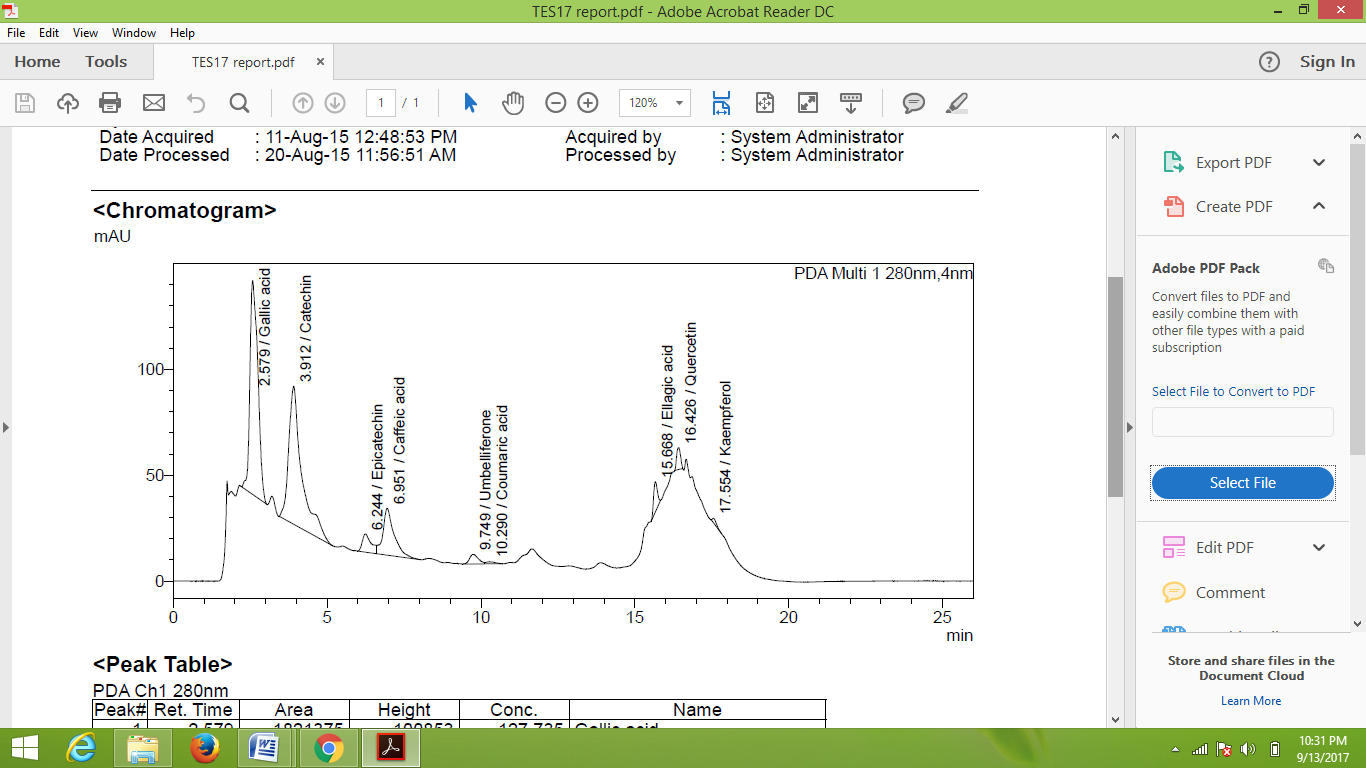


**Additional File 3:** UPLC analyses of TES17 extract showing nine different phenolic compounds based on particular retention time.
